# Perioperatives Management bei der Versorgung mit aktiven Rhythmusimplantaten

**DOI:** 10.1007/s00399-023-00989-6

**Published:** 2024-01-30

**Authors:** Konstantin Krieger, Innu Park, Till Althoff, Sonia Busch, K. R. Julian Chun, Heidi Estner, Leon Iden, Tilman Maurer, Andreas Rillig, Philipp Sommer, Daniel Steven, Roland Tilz, David Duncker

**Affiliations:** 1 Klinik für Kardiologie, Asklepios Klinikum Harburg, Eißendorfer Pferdeweg 52, 21075 Hamburg, Deutschland; 2 Klinik für Kardiologie u. Angiologie, Charite – Universitätsmedizin Medizin Berlin, Berlin, Deutschland Charitéplatz 1, 10117; 3 Abteilung für Elektrophysiologie, Herz-Zentrum Bodensee, Konstanz, Deutschland Luisenstraße 9A, 78464; 4 Cardioangiologisches Centrum Bethanien – CCB, Frankfurt am Main, Deutschland Im Prüfling 23, 60389; 5 Medizinische Klinik und Poliklinik I, LMU Klinikum der Universität München, München, Deutschland Ziemssenstraße 5, 80336; 6 Klinik für Kardiologie, Herz- und Gefäßzentrum Bad Segeberg, Bad Segeberg, Deutschland Am Kurpark 1, 23795; 7 Klinik für Kardiologie, Asklepios Klinik St. Georg, Hamburg, Deutschland Lohmühlenstraße 5, 20099; 8 Universitäres Herz- und Gefäßzentrum Hamburg, Universitätsklinikum Eppendorf Hamburg, Hamburg, Deutschland Martinistraße 52, 20251; 9 Med. Klinik für Elektrophysiologie/Rhythmologie, Herz- und Diabeteszentrum NRW, Ruhr-Universität Bochum, Bad Oeynhausen, Deutschland Georgstraße 11, 32545; 10 Abteilung für Elektrophysiologie, Herzzentrum der Uniklinik Köln, Köln, Deutschland Kerpener Straße 62, 50937; 11 Klinik für Elektrophysiologie, Medizinische Klinik II, Universitäres Herzzentrum Lübeck, Universitätsklinikum Schleswig-Holstein (UKSH), Lübeck, Deutschland Ratzeburger Allee 160, 23562; 12 Hannover Herzrhythmus Centrum, Klinik für Kardiologie und Angiologie, Medizinische Hochschule Hannover, Hannover, Deutschland Carl-Neuberg-Straße 1, 30625; 13 Arrhythmia Section, Cardiovascular Institute (ICCV), CLÍNIC – University Hospital Barcelona, Barcelona, Spanien C. de Villarroel, 170, 08036

**Keywords:** Kardiale Resynchronisationstherapie, Defibrillator, Herzschrittmacher, Perioperative Planung, Ambulantisierung, Cardiac resynchronization therapy, Defibrillator, Pacemaker, Perioperative planning, Outpatient surgery

## Abstract

Die Implantation aktiver Herzrhythmusimplantate („cardiovascular implantable electronic device“, CIED) stellt einen relevanten Teil der modernen Kardiologie dar, und eine sorgfältige perioperative Planung dieser Eingriffe ist notwendig. Präoperativ müssen alle Informationen vorliegen, die für die Indikation, den Eingriff und die Aufklärung relevant sind. Dies stellt die Basis für eine adäquate Geräteauswahl dar. Von entscheidender Bedeutung ist die Vorbeugung von Infektionen, u. a. durch die präoperative Gabe von Antibiotika und den perioperativen Umgang mit der Antikoagulation. Nach erfolgter Operation sind eine postoperative Überwachung, Systemkontrolle und apparative Diagnostik vor der Entlassung erforderlich. Die zunehmende Ambulantisierung erfordert eine Anpassung dieser Prozesse. Die vorliegende Übersichtsarbeit fasst das perioperative Management anhand praktischer Überlegungen zusammen.

## Abstract (en)

Cardiovascular implantable electronic devices (CIED) are an important part of modern cardiology and careful perioperative planning of these procedures is necessary. All information relevant to the indication, the procedure, and the education of the patient must be available prior to surgery. This provides the basis for appropriate device selection. Preoperative antibiotic prophylaxis and perioperative anticoagulation management are essential to prevent infection. After surgery, postoperative monitoring, telemetric control, and device-based diagnostics are required before discharge. These processes need to be adapted to the increasing trend towards outpatient care. This review summarises perioperative management based on practical considerations.

Im Jahr 2021 wurden in Deutschland etwa 98.000 Herzschrittmacher (SM) und etwa 37.700 Defibrillatoren (ICD) implantiert [1]. Aktive Herzrhythmusimplantate („cardiovascular implantable electronic device“, CIED) sind damit ein wesentlicher Bestandteil der modernen Kardiologie. Der perioperativen Planung eines CIED-Eingriffs kommt dabei eine zentrale Bedeutung zu. Eine Unterteilung in präoperative Planung, operative Durchführung und postoperative Nachsorge ist sinnvoll.

## Präoperative Planung

Zunächst ist im Rahmen der präoperativen Planung die Indikation für den geplanten Eingriff zu reevaluieren. Neben der Anamneseerhebung ist die körperliche Untersuchung und eine Sichtung der Vorbefunde (bei Revisionsoperation z. B. OP-Berichte von vorangegangenen CIED-Eingriffen) der Patienten unerlässlich. Die Händigkeit des Patienten zur Entscheidung über eine mögliche kontralaterale Implantation sollte erfragt werden. Zusätzliche anamnestische Überlegungen zur präoperativen Planung sind in Tab. 1 dargestellt.

**Table 1:** 

Anamnese	Fragestellung	Relevanz
**Freizeitaktivitäten**	*Statische Beanspruchung* Sportschütze/Jäger Geigenspieler	Platzierung des Aggregats
	*Dynamische Beanspruchung* Ruderer, Schwimmer Gewichtheben (Fitness)	Zugangswege
**Berufsanamnese**	Berufskraftfahrer	Einschränkung der Fahreignung
	Berufsumfeld (elektromagnetische Felder)	Inadäquate Schockabgaben Magnetreaktion
***Komorbiditäten***		
**Diabetes mellitus**	Wundheilungsstörungen Insulinpflichtigkeit	Wundpflege – Visiten Perioperative Hypoglykämie
**Niereninsuffizienz**	Terminale Niereninsuffizienz Dialyseshunt/-katheter	Anpassung an Dialyseschema Kontralaterale Operationsseite Infektionsrisiko Konstrastmittelnephropathie
**Chronisch Obstruktive Lungenerkrankung**	Sauerstofftherapie Lungenemphysem	O_2_-Gabe, Hyperkapniegefahr Pneumothoraxgefahr
**Onkologische Erkrankungen**	Mammakarzinom Portsystem Thorakale Radiatio Weitere Diagnostik	Lymphödem Operationsseite Aggregatlage
**Neurologische Erkrankung**	Demenzielle Erkrankung Morbus Parkinson	Compliance und Wundpflege Mobilität und Sturzneigung Schrittmacherinterferenzen
***Medikation***		
	Antithrombotische Therapie Insulintherapie Metformin SGLT2-Inhibitoren	Blutungen, Taschenhämatome Perioperative Hypoglykämie Laktatazidose Ketoazidose
***Kardiologische Vorerkrankungen***		
**Klappenvitien**	Trikuspidalklappen(TK)-Erkrankung Geplante/erfolgte TEER der TK TK-Rekonstruktion	Klappenpassage
**Herzchirurgische Eingriffe**	Mechanische Herzklappe	Erhöhte Blutungsgefahr – erschwertes Management Antikoagulation
**Herzrhythmusstörungen**	Permanentes Vorhofflimmern Vorhofablationen	Verzicht auf atriale Elektrode Atriale Elektrodenwerte
**Allergien**	Antibiotikaallergie Kontrastmittelallergien – Silikone, Titanium	Umstellung Antibiotika Gabe einer Allergieprophylaxe Alternative Beschichtung

**Figure 1:**
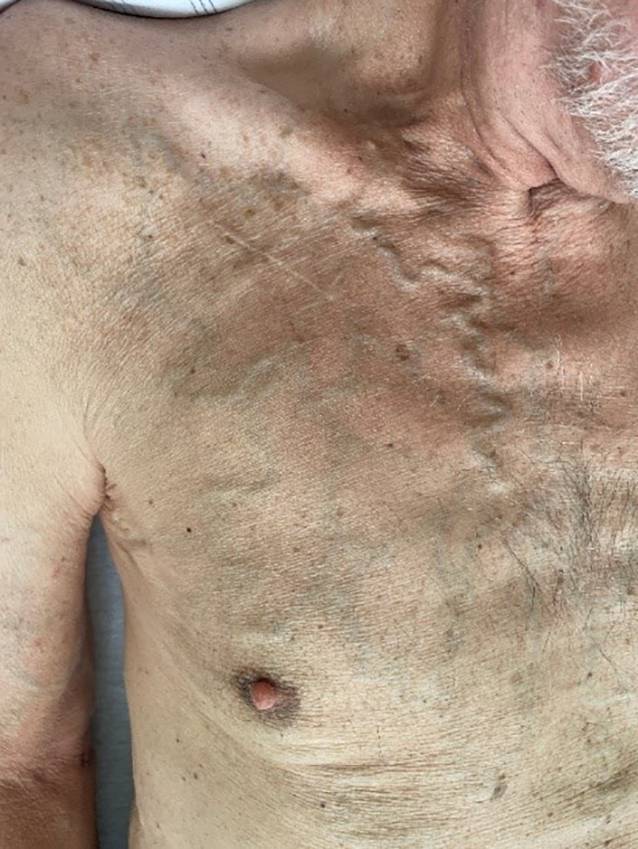

Eine Inspektion des Operationsgebiets, besonders im Rahmen von Revisionseingriffen, ist unerlässlich. Die Haut sollte intakt und frei von Infektionen sein. Narben können auf Voroperationen, wie z. B. eine Port- oder Bypassoperation hinweisen. Eine vermehrte Gefäßzeichnung kann eine Stenose oder einen Verschluss des venösen Abstromgebiets signalisieren, weshalb insbesondere vor Revisionseingriffen oder Aufrüstungen präoperativ eine Phlebographie durchgeführt werden sollte (Abb. 1). Die relevante präoperative apparative Diagnostik ist in Tab. 2 zusammengefasst.

**Table 2:** 

Diagnostik	Befund	Relevanz
*Obligat*		
**Vitalwerte**	Körpertemperatur (Fieber innerhalb 24 h [2])	Infektionsrisiko
**Elektrokardiogramm (EKG)**	Sinusrhythmus oder Vorhofflimmern (Links‑)Schenkelblock – QRS-Breite (> 130 ms bzw. ≥ 150 ms)	Notwendigkeit einer atrialen Elektrode CRT-CSP-Indikation prüfen
**Transthorakale Echokardiographie (TTE)**	Linksventrikuläre Ejektionsfraktion Größe der Herzkammern Klappenvitien	CRT-CSP/ICD-Indikation prüfen Elektrodenplatzierung Klappenpassage
**Labor**	Gerinnungswerte (INR, aPTT), Thrombozytenzahl Elektrolyte Nierenretentionswerte (eGFR) Infektwerte (CRP/Leukozytenzahl) Hämoglobin	Blutungsgefahr Herzrhythmusstörungen Kontrastmittelgabe Ausschluss Infektion Anämie
*Fakultativ*		
**Langzeit-EKG**	Sinusknotenfunktion AV-Überleitungsstörungen	Aktivierung und Art des Sensors AV-Suchhysterese
**Röntgen-Thorax**	Anatomische Gegebenheiten Emphysemthorax Anzahl, Lage und Integrität der Elektroden bei Revisionseingriffen Konnektoren	Operative Zugangswege Stillgelegte Elektroden Lage des Aggregates Pneumothoraxgefahr
**Belastungs-EKG**	Chronotrope Inkompetenz AV-Überleitungsstörungen	Programmierung und Art des Sensors AV-Suchhysterese
**Phlebographie**	Verschluss der Zugangswege	Kontralateraler Zugang Explantation
**Abfrage des Aggregats**	Batteriestatus Elektrodenwerte Gespeicherte Arrhythmien	Aggregatwechsel Elektrodenrevision Aufrüstung auf (CRT-)ICD

### Aufklärung

Eine schriftliche Aufklärung muss im adäquaten zeitlichen Abstand zum operativen Eingriff erfolgen. Unumstritten ist, dass eine frühzeitige Aufklärung es ermöglicht, offene Fragen rechtzeitig zu klären. Die relevanten Komplikationen mit ihren Häufigkeiten sind in Tab. 3 zusammengefasst. Ferner sind gesetzlich versicherte Patienten über den Anspruch auf Einholung einer Zweitmeinungen aufzuklären [3].

**Table 3:** 

Komplikation Inzidenz	(%)
Prozedurbedingte Mortalität	≤ 0,1
30-Tage-Mortalität	0,6–1,4
Taschenhämatom	0,2–16
Infektion	0,6–3,4
Sondendislokation	1,2–3,3
Pneumothorax	0,4–2,8
Perikardtamponade	0,5–1,5
Klinisch bedeutsame Perforation	0,1–1,5
Andere	< 0,5

#### Blutungen

Blutungskomplikationen sind bei CIED-Eingriffen mit 0,2–16 % [4] die häufigste Komplikation, weshalb das perioperative Management der antithrombozytären Therapie von entscheidender Bedeutung ist. Die perioperative Fortsetzung einer bestehenden Therapie mit Vitamin-K-Antagonisten (VKA; [5]) oder NOAC [6] führt zu einer signifikanten Reduktion von Hämatomen gegenüber einem Bridging mit Heparin. Die perioperative Fortsetzung einer Thrombozytenaggregation kann in Einzelfällen 3 bis 7 Tage vor der Operation pausiert werden (Tab. 4; [4]). Grundsätzlich sollten nichtdingliche Eingriffe verschoben werden, bis die reguläre Dauer der dualen Thrombozytenaggregationshemmung beendet werden kann. Bei Patienten mit erhöhtem Blutungsrisiko kann ein Druckverband oder Sandsack hilfreich sein, um die Ausbildung eines relevanten Hämatoms zu vermeiden. Dieser sollte für max. 24 h angewendet werden. Entscheidend ist, dass durch die Verhinderung von Blutungskomplikationen das Risiko für eine CIED-Infektion signifikant reduziert wird [7].

**Table 4:** 

Orale Antikoagulation (OAC)		OAC plus P2Y_12_	Duale Plättchenaggregationshemmung (DAPT)	
**NOAK**	**VKA**	OAC weiter! (VKA oder NOAK) Pause P2Y_12_-Inhibitoren abhängig vom Patienten spezifischen Risiko	**PCI bei ACS oder andere hohe Ischämierisiken**^**a**^	
**Fortsetzung** **(ggf. 1–2 Dosen pausieren)**	Fortsetzung (INR abhängig von Indikation, < 3,5)		Nein	Ja
			**<** **1 Monat**	**<** **6 Monate**
*Alternativ*			Fortsetzung DAPT	Fortsetzung DAPT (evtl. Pause P2Y_12_-Inhibitoren)
**Pause abhängig von CrCL und NOAK**	Pause *ohne* Heparin-Bridging (CHA_2_DS_2_-VASc Score < 3)		**>** **1 Monat**	**>** **6 Monate**
			ASS fortsetzen, evtl. Pause P2Y_12_-Inhibitoren	Pausierung P2Y_12_-Inhibitoren

^a^Z. n. Stentthrombose trotz APT, PCI des letzten verbliebenen Koronargefäßes, diffuse Mehrgefäßerkrankung bei Diabetes mellitus, CrCL < 60 ml/min, ≥ 3 Stents, Bifurkation mit 2 Stents, totale Stentlänge > 60 mm, CTO-Intervention

*NOAK* neue orale Antikoagulanzien, *PCI* perkutane Koronarintervention, *CrCl* Kreatinin-Clearance, *VKA* Vitamin-K-Antagonist

#### CIED-Infektionen

Die Patienten müssen präoperativ über das Risiko einer frühen oder späten CIED-Infektion aufgeklärt werden. Zweiteingriffe, vor allem ICD- oder CRT-assoziierte Eingriffe haben ein höheres Infektionsrisiko [8]. Auch die patientenassoziierten Faktoren haben einen Einfluss auf das Auftreten einer CIED-Infektion (Infobox 1). Eine Zusammenfassung der wichtigsten Punkte zur Verhinderung dieser Faktoren wird in Infobox 2 gegeben [4, 9].

#### Infobox 1 Risikofaktoren für CIED-Infektionen

- *Patientenassoziierte Risiken: *Z. n. Endokarditis, dialysepflichtige Niereninsuffizienz, Diabetes mellitus
- *Eingriffsassoziierte Risiken: *Reoperation, Revision, Aggregegatwechsel
- *Operative Konsequenzen: *sondenloser SM, subkutaner oder extravaskulärer ICD, antibakterielle Hülle

#### Infobox 2 Maßnahmen zur Vermeidung von CIED-Infektionen

**„Dos“:**

- Antibiotikaprophylaxe innerhalb von 1 h vor Inzision
- Haarentfernung mit elektrischem Clipper (am Operationstag)
- Chirurgische Vorbereitung mit alkoholischem Chlorhexidin
- Spülung der Wunde mit sterilem NaCl
- Steriler Verband für 2–10 Tage

**„Don’ts“:**

- CIED-Implantationen bei akuten Infektionen (< 24 h Fieber)
- Instillation von Antiseptika und Antibiotika in die CIED-Tasche
- Verwendung von geflochtenem Nahtmaterial für den finalen Hautverschluss
- Routinemäßige postoperative Antibiotikatherapie
- Temporäre transvenöse Schrittmacher und zentrale Venenkanüle

#### Elektrodenkomplikationen

Die häufigsten Komplikationen, die zu einer Reoperation führen, sind frühe Sondendislokationen (2,4 %; [10]), insbesondere Dislokationen der LV-Elektrode im Rahmen von CRT-Eingriffen. Eine Abnahme der Reizschwelle oder ein Verlust der Wahrnehmung können genauso wie ein Isolationsdefekt oder Elektrodenbrüche im Langzeitverlauf zu einer Revision führen und sollten in der Aufklärung erwähnt werden.

#### Pneumo- und Hämatothorax

Die V. cephalica oder die V. axillaris sollte der bevorzugte Zugangsweg sein [11]. Die Punktion der V. subclavia lässt sich nicht immer vermeiden, gerade bei einem Revisions- oder CRT-Eingriff, so dass Komplikationen wie ein Pneumo- oder Hämatothorax sowie eine späte Sondendysfunktion durch ein Subclavian-Crush-Syndrom nicht gänzlich auszuschließen sind. Die ultraschallgestützte Punktion der V. axillaris stellt eine sichere Alternative zur Punktion der V. subclavia dar [12] und zeigt im Langzeitverlauf eine Reduktion der Komplikationen gegenüber der Punktion der V. subclavia [13]. Diese möglichen Komplikationen müssen mit den Patienten kommuniziert werden.

#### Fahreignung

Die Patienten sollten bereits im Rahmen des Aufklärungsgesprächs über eine mögliche Einschränkung der Fahreignung über einen gewissen postoperativen Zeitraum aufgeklärt werden. Wir verweisen auf die Begutachtungsleitlinien zur Kraftfahreignung [14].

#### Aufklärung vor ICD-Implantation

Patienten, die einen ICD oder CRT‑D erhalten, sei es aus primär- oder sekundärprophylaktischer Indikation, sollten gesondert über die höheren Komplikationsraten informiert werden [8]. In diesem Zusammenhang sollte den Patienten auch das Vorgehen nach einem ICD-Schock und die Möglichkeit adäquater, aber auch inadäquater Schocks erläutert werden.

### Eingriffsspezifische Fragestellungen

**Figure 2:**
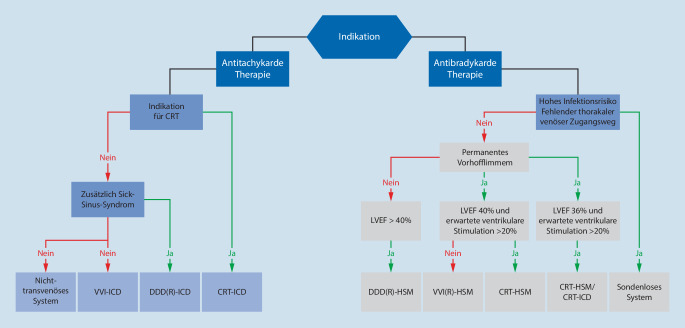

Abhängig von der Indikation sollte präoperativ festgelegt werden, welches CIED die Patienten benötigen. Ein entsprechendes Flussschema zur Entscheidung, angelehnt an die Empfehlungen der ESC [15] ist in Abb. 2 dargestellt.

Vor einem geplanten Eingriff sind bei der Wahl der Elektroden und des Aggregats einzelne Punkte zu beachten, die in Tab. 5 genauer erläutert werden.

**Table 5:** 

Anteil	Option	Vorteil	Hinweis
***Elektrode***			
**Vorhofelektrode**	*Aktive* vs. passive Fixierung	Geringere Dislokationsrate	Evtl. Platzierung am Bachmannbündel
**Ventrikelelektrode**	*Aktive* vs. Passive Fixierung	Septale Platzierung	Evtl. Physiologische Stimulationsstelle
**ICD-Konnektor**	*DF4* vs. DF1	Weniger Konnektoren	Wichtig zu wissen vor Aggregatwechsel oder Revision
**ICD-Elektrode**	*Single* vs. Dual Coil	Einfachere Explantation	Dual Coil kann bei rechtsseitiger Implantation erwogen werden
**Linksventrikuläre Elektrode**	*Quadripolare* vs. bipolare Elektrode	Basale Stimulation Verhinderung Phrenikusstimulation	Wichtig zu wissen vor Aggregatwechsel oder Revision!
***Aggregat***			
**Bewegungssensor**	AMV-Sensor Erschütterungssensor	Verbesserung der chronotropen Kompetenz	Abhängig vom Alter und Aktivität der Patienten
**Telemonitoring**	Ja vs. nein	Reduktion der ambulanten Besuche Erhöhte Sicherheit	Vorrangig bei ICD oder Herzinsuffizienzpatienten

*AMV* Atem-Minuten-Volumen, *MRT* Magnetresonanztomographie

Die kursive Option stellt die empfohlene Wahl dar

Insbesondere vor einem geplanten Aggregatwechsel, der häufig ambulant durchgeführt wird und bei dem die Gefahr besteht, dass dem Operateur nicht alle relevanten Vorbefunde vorliegen, sollte eine aktuelle Echokardiographie mit Bestimmung der linksventrikulären Funktion vorliegen. Denn in der Zwischenzeit kann sich eine Schrittmacher-induzierte Herzinsuffizienz entwickelt haben, die im Rahmen des geplanten Aggregatwechsels durch eine Aufrüstung auf eine biventrikuläre oder eine Stimulation des Reizleitungssystems (Conduction System Pacing / CSP) mitbehandelt werden kann. Das CSP gewinnt zunehmend an Bedeutung, insbesondere im Rahmen der Herzinsuffizienztherapie und bei frustranen Implantationen von linksventrikulären Elektroden. Die perioperative Vorbereitung ist vergleichbar mit der konventionellen CIED-Therapie, auf eine detaillierte Erläuterung wird in dieser Arbeit verzichtet.

### Strukturelle Voraussetzungen

Vor der Planung des Eingriffs sollte geklärt sein, dass die räumlichen und apparativen Gegebenheiten für die Durchführung eines CIED-Eingriffs geeignet sind [16]. Eine entsprechende Raumlufttechnik muss vorhanden und die hygienischen Voraussetzungen müssen gegeben sein. Grundsätzlich können alle CIED-Eingriffe in (Hybrid‑)Operationssälen durchgeführt werden, sofern diese die entsprechende Lüftungsklasse erfüllen. Eine enge Abstimmung mit der Hygiene sollte erfolgen. Personen, die im Zusammenhang mit der Anwendung von Röntgenstrahlen tätig werden, insbesondere solche, die Zugang zum Kontrollbereich (Röntgenraum) haben, müssen gemäß § 63 StrlSchV vor Aufnahme der Tätigkeit und danach jährlich über die Arbeitsverfahren, die anzuwendenden Schutzmaßnahmen und mögliche Gefahren unterwiesen werden. Insgesamt sollten die Operateure und das Assistenzpersonal die entsprechende Qualifikation und Erfahrung haben, um mögliche Komplikationen beherrschen zu können [15]. Neben der Möglichkeit zur Überwachung der Vitalwerte sollten im Operationssaal Notfallausrüstung wie Defibrillator und Notfallmedikamente (Adrenalin, Atropin, Isoprenalin etc.) verfügbar sein [4].

## Operative Planung

**Figure 3:**
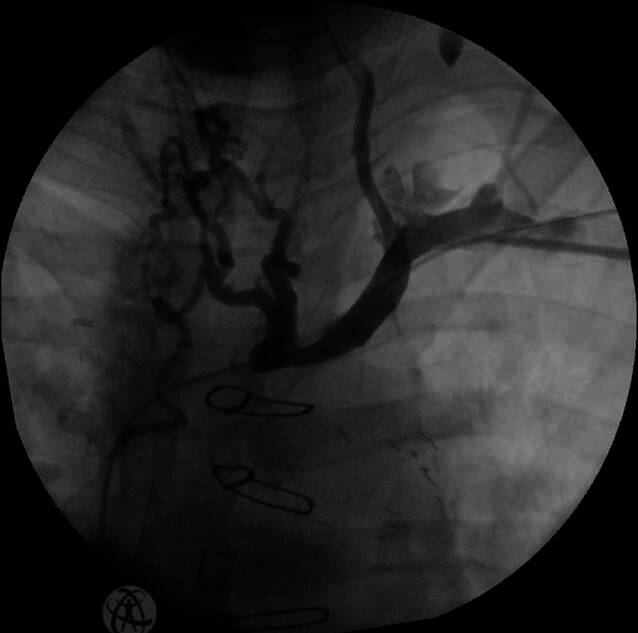

Ein Team-Time-out-Bogen hilft Fehler zu vermeiden. Direkt vor der Operation sollte eine Patientenvisite durchgeführt werden, um die persönlichen Daten, die Nüchternheit und die Medikamenteneinnahme bzw. -pause zu überprüfen. Außerdem sollten die Patienten u. a. bzgl. Fieberfreiheit (> 24 h) befragt werden. Vor der Implantation sollte ein peripher-venöser Zugang gelegt werden, um Medikamente verabreichen zu können. Dabei ist ein zum Operationsgebiet ipsilateraler Venenzugang sinnvoll (z. B. V. mediana cubiti), um ggf. frühzeitig eine Phlebographie zur Darstellung des V. axillaris/subclavia-Systems durchzuführen. Da die linke Seite bei den meisten Patienten die nichtdominante Seite in Bezug auf die Händigkeit darstellt und bei ICD-Therapien eine geringere Defibrillationsschwelle (DFT; [4]) zeigt, sollte diese Seite der bevorzugte Zugangsweg sein (Abb. 3).

### Operative Überwachung

Nach einer adäquaten Lagerung der Patienten erfolgt die Anlage der Monitorüberwachung. Diese sollte aus einer Überwachung von EKG, Blutdruck- und Sauerstoffsättigung bestehen, wobei letztere über eine akustische Rückmeldung verfügen sollte, um einen Abfall von Sauerstoffsättigung und Puls frühzeitig zu erkennen. Zusätzlich sollten bei Patienten mit ICD-Eingriffen und ggf. bei SM-Eingriffen Defibrillationspatches aufgeklebt und ein externer Defibrillator mit der Möglichkeit zur transkutanen Stimulation angeschlossen werden.

### Antibiotikagabe

Eine präoperative Antibiotikagabe zur Verhinderung von Infektionen sollte innerhalb von 1 h vor Inzision erfolgen. Bevorzugtes Antibiotikum ist Cefazolin 1–2 g i.v. oder Flucloxacillin 1–2 g i.v., bei Allergie oder erhöhtem Risikoprofil für resistente Keime alternativ Vancomycin (15 mg/kgKG, dann innerhalb 90–120 min; [15]). Eine postoperative Antibiose wird nicht routinemäßig empfohlen.

### Analgosedierung

Eine präoperative Analgesie z. B. mit Morphin (3–5 mg i.v.) kann unabhängig von der Art der Lokalanästhesie (z. B. mit Xylocain oder Lidocain) in Kombination mit einer antiemetischen Medikation (z. B. Ondansetron 4 mg i.v.) zu einer deutlichen Schmerzreduktion führen. Obwohl Morphin sicherlich auch eine sedierende Komponente hat, kann zur weiteren Sedierung oder Anxiolyse eine Medikation mit Midazolam erwogen werden. Zu beachten sind jedoch – insbesondere bei älteren Patienten – die damit verbundenen möglichen paradoxen Reaktionen bzw. das Auftreten einer Atemdepression. Sinnvoll erscheint dann eine kontinuierliche Sauerstoffinsufflation von 1–2 Litern über eine Nasenbrille. Eine tiefere Analgosedierung mit kontinuierlicher Gabe von Propofol oder Remifentanyl ist ebenfalls möglich. Zur Durchführung einer Analgosedierung und zur Beherrschung möglicher Anpassungen und Komplikationen sollte das ärztliche und Assistenz-Personal entsprechend geschult und die Patienten dementsprechend aufgeklärt sein. Die Analgosedierung ist eine separate medizinische Leistung und bedarf daher auch einer separaten Aufklärung [17]. Die Art und Intensität der Sedierung müssen vor allem auch vor dem Hintergrund potenziell ambulant durchzuführender Prozeduren (adäquate postoperative Überwachung!) sorgfältig ausgewählt und titriert werden.

### Desinfektion des Operationsgebiets

Als präoperatives Hautantiseptikum für allgemeine chirurgische Operationen und intravaskuläre Katheterisierungen zeigten randomisierte Studien eine Überlegenheit bei Verwendung von alkoholhaltigen 2 %-Chlorhexidin-Lösungen (zunehmend durch Octenidinhydrochlorid ersetzt) im Vergleich zu Iod-Povidon-Lösungen [9]. Allerdings gibt es bis dato keine randomisierten Daten zur Anwendung bei CIED-Eingriffen. Wichtig ist vor allem die ausreichende Einwirk- (Octenidin 60–120 s) und die Trockenzeit vor Inzision.

## Postoperative Nachsorge

Unabhängig davon, ob es sich um einen ambulanten oder stationären Eingriff handelt, ist eine weitere Überwachung indiziert. Je nach Art des Eingriffs und des damit verbundenen Risikos sollte eine 1‑ bis 4‑stündige Überwachung erfolgen, außer bei Sondenextraktionen, bei denen eine 12-stündige Überwachung angezeigt ist [18].

### Röntgen-Thorax

Vor der Entlassung sollte eine Röntgenuntersuchung des Thorax in 2 Ebenen zum Ausschluss eines Pneumothorax und Dokumentation der Elektrodenlage erfolgen. Eine Vergleichsaufnahme kann bei Elektrodenproblemen wie V. a. Dislokationen helfen, das Problem frühzeitig zu erkennen.

### CIED-Abfrage

Vor der Entlassung müssen eine ärztliche Visite und eine Kontrolle des implantierten Systems erfolgen. Es sollte eine individualisierte Programmierung entsprechend der Implantationsindikation und die Aktivierung möglicher Zusatzfunktionen erfolgen. Das weitere Verband- und Wundmanagement in Abhängigkeit von der Hautnaht sowie eine zeitnahe Wiedervorstellung bei Beschwerden oder Auffälligkeiten im Wundbereich sollten besprochen werden. Eine komplette Ruhigstellung des Arms auf der Eingriffsseite sollte nicht erfolgen [4]. Die Patienten müssen vor der Entlassung einen Implantatausweis erhalten, erneut über eine mögliche Einschränkung der Fahreignung aufgeklärt werden und erhalten einen Termin zur Nachsorge innerhalb von 2–12 Wochen [15].

### Patienteninformation

Eine ausführliche Information für Patienten vor Entlassung, idealerweise auch schriftlich, bezüglich Verhaltensmaßnahmen scheint eine sinnvolle Möglichkeit, postoperative Verhaltensempfehlungen zu vermitteln und den Patienten eventuelle Unsicherheiten zu nehmen.

## Ambulantisierung

Seit dem MDK-Reformgesetz ist nach § 115b SGB V Abs. 1A eine Erweiterung des Katalogs zum ambulanten Operieren im Krankenhaus (AOP) erfolgt. Diese wurde zum 01.01.2023 vorgelegt und gilt nach einer Übergangsfrist seit dem 01.04.2023. Danach sollen kardiologische Eingriffe, v. a. Herzschrittmacher–Implantationen, aber auch ICD-Aggregatwechsel verstärkt ambulant durchgeführt werden. Um Leistungen des AOP-Katalogs stationär abrechnen bzw. Patienten stationär aufnehmen zu können, wurden die bisherigen G‑AEP-Kriterien durch Kontextfaktoren ersetzt. Dabei werden die Komorbiditäten durch komplexe Scores berechnet und fast nur Akuterkrankungen in die Berechnungen einbezogen. Aufgrund dessen ergeben sich abweichende Empfehlungen der DGK zur ambulanten Durchführbarkeit von CIED-Eingriffen und den Vorgaben des aktuellen AOP-Katalogs [18]. Patienten haben kein Mitspracherecht über die ambulante oder stationäre Durchführung des Eingriffs im Sinne eines „shared decision making“. Nichtsdestotrotz müssen CIED-Eingriffe auf die Möglichkeit einer ambulanten Durchführbarkeit überprüft werden, und dies sollte mit den Patienten im Rahmen der Vorbereitung geklärt werden. In diesem Zusammenhang müssen im implantierenden Zentrum eine Struktur und das Personal vorhanden sein, um die unter dem Punkt postoperative Nachsorge genannten Punkte rechtzeitig vor der Entlassung aus dem Krankenhaus durchführen zu können. Es muss eine Struktur geschaffen werden, die den Patenten im Notfall eine Vorstellung im eigenen oder einem kooperierenden Zentrum vorhält. Im Vordergrund muss jedoch immer die Patientensicherheit stehen und die Möglichkeit, einen ambulanten Fall in einen stationären Fall umzuwandeln, um die Patienten stationär aufnehmen zu können. Dies muss unabhängig von wirtschaftlichen Überlegungen der Leistungserbringung geschehen.

## Fazit für die Praxis

- Eine gute perioperative Planung hilft, Behandlungsfehler zu vermeiden, Komplikationen zu minimieren sowie die Patientensicherheit und -zufriedenheit zu erhöhen.
- Zur Verringerung des Infektionsrisikos ist die Vermeidung von Blutungskomplikationen essenziell.
- Insbesondere im Hinblick auf die zunehmende Ambulantisierung sollten Strukturen zur postoperativen Betreuung der Patienten etabliert werden.

## Abkürzungen

AMV: Atem-Minuten-Volumensensor
APT: Antithrombozytäre Plättchenhemmung
CIED: Kardiale implantierbare elektrische Rhythmusimplantate
COPD: Chronisch-obstruktive Lungenerkrankung
CRT: Kardiale Resynchronisationstherapie
CSP: Conduction system pacing
DAPT: Duale antithrombozytäre Plättchenhemmung
DGK: Deutsche Gesellschaft für Kardiologie
EHRA: European Heart Rhythm Association
ESC: European Society of Cardiology
ICD: Implantierbarer Kardioverter-Defibrillator
MRT: Magnetresonanztomographie
NOAC: Neue orale Antikoagulanzien
OSAS: Obstruktives Schlafapnoe Syndrom
S‑ICD: Subkutan implantierbarer Kardioverter-Defibrillator
SM: Herzschrittmacher
TEER: Transcatheter edge-to-edge repair
VKA: Vitamin-K-Antagonist
